# Same but different? A thematic analysis on adalimumab biosimilar switching among patients with juvenile idiopathic arthritis

**DOI:** 10.1186/s12969-019-0366-x

**Published:** 2019-10-04

**Authors:** William D. Renton, Helen Leveret, Catherine Guly, Heather Smee, Jamie Leveret, Athimalaipet V. Ramanan

**Affiliations:** 1 Bristol Royal Hospital for Children, University Hospitals Bristol NHS Foundation Trust, Bristol, UK; 2 Bristol Eye Hospital, University Hospitals Bristol NHS Foundation Trust, Bristol, UK; 3 Translational Health Sciences, Bristol Medical School, University of Bristol, Bristol, UK

**Keywords:** Paediatric rheumatology, Juvenile idiopathic arthritis, Uveitis, Biosimilars, Adalimumab, Qualitative

## Abstract

**Background:**

Biologic medications have dramatically enhanced the treatment of many chronic paediatric inflammatory conditions. Their high cost is a factor that prohibits their broader use. Cheaper generic versions, or biosimilars, are increasingly being used. Healthcare services are switching some patients over to biosimilars for economic reasons, known as ‘non-medical switching’. Some patients unsuccessfully switch due to perceived decreases in efficacy or non-specific drug effects. The implications of failed switching include exhaustion of therapeutic options, unnecessary exposure to other medications, increased healthcare utilisation, worse patient outcomes and higher overall healthcare costs. Patient perceptions almost certainly play a role in these ‘failed switches’.

**Methods:**

A thematic analysis was performed to better understand patient and parent perceptions on non-medical biosimilar switching. The study was conducted in accordance with the Consolidated Criteria for Reporting Qualitative Research recommendations. Patients with juvenile idiopathic arthritis currently taking adalimumab were included.

**Results:**

Nine families were interviewed just prior to a hospital trust-wide non-medical switch to an adalimumab biosimilar. Several common themes were identified. The most frequent concerns were regarding practical aspects of the switch including the medication administration device type; the colour of the medication and administration device; and whether the injections would sting more. The relative safety and efficacy of the biosimilar was raised although most families felt that there would be no significant difference. Anxieties about the switch were largely placated by reassurances from the medical team.

**Conclusions:**

We derived recommendations based on existing adult literature and the observations from our study to optimise the benefits from non-medical biosimilar switching.

**Electronic supplementary material:**

The online version of this article (10.1186/s12969-019-0366-x) contains supplementary material, which is available to authorized users.

## Background

Biologic medications, including monoclonal antibodies, are medications derived from living organisms. These medications, including adalimumab, have dramatically improved outcomes of chronic inflammatory conditions including refractory juvenile idiopathic arthritis (JIA) [1, 2] and JIA associated uveitis [3, 4].

Biologics are expensive and their cost is a factor that prohibits their broader use. Many index biologics (bio-originators) are still subject to copyright patents, contributing to their high cost. However, for several biologics, generic versions (biosimilars) are becoming available. Unlike conventional medications, biosimilars are not considered completely equivalent to their bio-originator as they are large and complex molecules that are very sensitive to any slight change in the manufacturing process [5].

Biosimilar developers must demonstrate that their biosimilar is highly similar to the bio-originator (notwithstanding normal variability inherent to all biologics) and that there are no clinically meaningful differences regarding quality, safety and efficacy [6, 7]. Regulating bodies, including the European Medicines Agency, and rheumatology groups have encouraged a Bayesian approach to the development of biosimilars in order to abbreviate licencing pathways, help lower costs and increase access to these medications [8–10]. Data for one indication may be extrapolated to others (assuming the same mechanism of action is used), again easing the statistical threshold and abbreviating the approval process [11, 12].

Theoretical concerns when switching to biosimilars include a loss of efficacy, changes in immunogenicity (including the development of anti-drug antibodies) and differences in the safety profile compared with the bio-originator [13]. Despite these apprehensions, outcomes from blinded, randomized, controlled trials in adults have been reassuring [14]. While this is the case, large scale paediatric trials are lacking**.** Nonetheless, healthcare services are tending towards switching patients to biosimilars for economic reasons, known as ‘non-medical switching’ [13].

Experience among adults suggests that the uptake of biosimilars in open label environments is hindered when compared to blinded trials. These ‘failed switches’ are usually attributed to subjective reports of perceived decrease in efficacy or non-specific drug effects [15–17]. These are thought to largely be due to the nocebo effect; noxious reactions to therapeutic interventions that occur because of negative expectations of the patient [18]. Emerging paediatric data, while scarce, suggests that some children also unsuccessfully switch [19]. The implications of failed switching could potentially include exhaustion of therapeutic options, unnecessary exposure to other medications, increased healthcare utilisation, worse patient outcomes and higher overall healthcare costs.

It is hypothesised that patient perceptions strongly influence failed biosimilar switching [20].

## Methods

This study aims to develop an understanding of the perceptions of paediatric patients and their parents with regard to biosimilar switching. A thematic analysis was performed. Patients with a diagnosis of JIA, under the age of 18 years, on adalimumab (a fortnightly subcutaneous injection) were included. All families were English speaking and literate. They were recruited from paediatric rheumatology outpatient clinics at the Bristol Children’s Hospital and Bristol Eye Hospital, tertiary hospitals in the United Kingdom, over a two-week period in December 2018. The study was performed prior to a trust-wide mandatory change from the adalimumab bio-originator to a biosimilar on guidance from the National Health Service (NHS).

Patients were invited to participate either by telephone (5 families) or at the time of clinical appointments (4 families). Convenience sampling was predominantly used with supplementary purposive sampling (aiming to include a range of patients representative of the typical distribution of age, gender and disease severity). Interviews were conducted in a private setting at the outpatient department on the same day as scheduled appointments if possible.

Two researchers with experience in qualitative research methods, but without expertise in paediatric rheumatology or biologic medications (HL and JL) ran the patient led, semi-structured interviews. They had not previously met the families. Participants understood the purpose of the study and that the interviewers were not part of the treating team, nor experts in paediatric rheumatology or biologics. Individual families were interviewed on a single occasion and had no further active participation in the study following the interviews. Patients were interviewed together with their parent(s). Questions were directed to all participants and specifically to the patient if they did not volunteer an answer. Prompting questions were used if required (Additional file 1). Families were provided a copy of a plain language summary outlining the process of transition to the biosimilar (designed by the trust pharmacy and paediatric rheumatology team) on the day of the interviews (Additional file 2).

Interviews were audiotaped and transcribed verbatim. Field notes were also taken during the interviews. Names and identifying features were removed from the transcripts for anonymity.

An inductive approach was taken; identifying and developing themes from the data [21, 22]. Coding and analysis were conducted by HL and JL using a qualitative analysis computer program (NVivo 12.2.0, QSR International). WR separately coded and analysed the data using the same program. Analyses were compared and integrated to create unifying themes.

Informed, written consent was obtained at the time of recruitment. The study was prospectively approved by the University Hospitals Bristol Questionnaire, Interview & Survey Group. The study was conducted and written in accordance with the Consolidated Criteria for Reporting Qualitative Research (COREQ) recommendations [23].

## Results

Of 10 families invited to participate, 9 consented and completed the interviews (1 declined due to time constraints). Eight interviews were conducted with the child and at least 1 parent contributing, while 1 interview was conducted with a patient’s parents only. All patients had a diagnosis of JIA with associated uveitis. Patient demographics are detailed in Table 1.

**Table 1:** Patient demographic details

Age	Gender	Time since diagnosis (months)	Time since commencing adalimumab (months)	Ocular complications	Active joint count at time of review	Uveitis activity at time of review *
6	F	37	24	Nil	0	Right 0 Left 0
8	F	72	13	Nil	0	Right 2+ Left 2+
10	M	66	58	Cataract, posterior synechiae, ocular hypertension	0	Right 0.5+ Left 0.5+
11	F	82	27	Nil	1	Right 0.5+ Left 0.5+
12	M	43	25	Cataract	0	Right 0.5+ Left 0.5+
12	F	127	80	Posterior synechiae	0	Right 0 Left 0
13	M	143	122	Cataract, posterior synechiae, band keratopathy	0	Right 0 Left 0.5+
15	F	172	21	Nil	0	Right 0.5+ Left 0.5+
17	F	190	59	Nil	0	Right 0.5+ Left 0.5+

*anterior chamber cell grading as per standardization of uveitis nomenclature (SUN) criteria [24]

Interviews lasted between 6 and 51 min (mean 17). All patients and parents involved made significant contributions to the interview answers. Most families raised some concerns but had an overall positive or apathetic view on the switch. One family was strongly dissatisfied about the non-optional change.

**Fig. 1:**
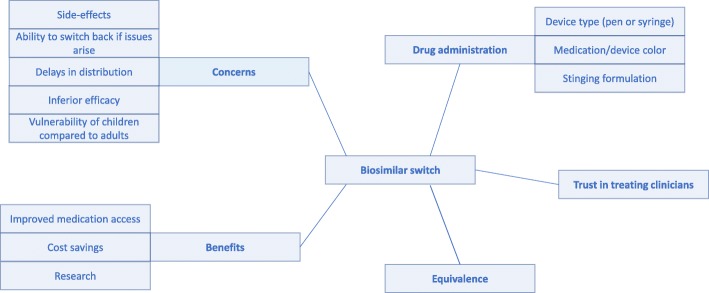
Thematic schema

The study identified five main themes concerning patients’ and families’ perceptions of the switch to a biosimilar medication; ‘drug administration, ‘concerns’, ‘benefits’, ‘equivalence’, and ‘trust in treating clinicians’. Some themes contained clear associated sub-themes outlined in the thematic schema (Fig. 1).

### Drug administration

Issues surrounding practicalities of drug administration were important to most respondents. These issues resonated with the patients more than other issues which their parents were more concerned about.

Patients were anxious about having access to the same medication administration device (the bio-originator is available as either a prefilled syringe or prefilled auto-injecting pen) and the potential of having to use an unfamiliar device.

*“I wasn't sure whether there's going to be an option of prefilled syringe and pen device … we are using a prefilled syringe … she said she would like to stick with that.” (parent)*

Past versions of the adalimumab bio-originator contained a citrate preservative which contributed to stinging as the medication is injected. Several patients recall what this felt like and those that have never been on a stingy preparation were aware of its existence. Whether the new medication would sting was a prime concern, although some patients were able to balance a possible worse outcome on an individual level against a perceived benefit for society.

*“Your concerns as well have been about ‘just tell me it's not going to hurt anymore’ or … about whether it's stingy or not.” (parent to patient)*

*“How would you feel about taking it if it did hurt? (interviewer) “I think it probably be good for society so I guess I would probably do it.” (patient)*

The colour of a biosimilar medication, administration device and its packaging was a major concern for most families. Several families specified concerns about the colour yellow (no other colours were specified), most attributing this to their previous experience with methotrexate.

*“ … if it is yellow, he's not going to go near it.” (parent)*

*“ [if] it is yellow and he can see it is yellow then that will make him gag because it is a psychological thing.” (parent)*

### Concerns

Within the theme of ‘concerns’, anxiety about side effects was frequently expressed. There was anxiety about switching from the known to the unknown. For other families, provided there were no new side effects, they didn’t have any significant concerns about switching to a biosimilar medication.

*“It's not ideal because you know that something works and it really is just to know that it's not going to have the side effects … .” (parent)*

*“We understand it, as long as he doesn't have any side effects detrimental to him.” (parent)*

Several families were concerned about what would happen if there were problems with the switch and whether it would be possible to switch back in the event of a poor outcome.

*“If we swap and it is worse, and it doesn't do anything, what happens then? Are we then stuck effectively with … the cheaper product?” (parent)*

Some were concerned about the logistics of switching to a biosimilar and whether the home delivery process would be disrupted, especially given the need to store these medications in a refrigerator.

*“My main concern is that the smooth transition goes through to [delivery service provider] because they are not always accurate with the delivery time.” (parent)*

There was some uncertainty about whether a biosimilar would be as effective as the bio-originator. Several patients referred to the fact that as it is cheaper, it may be inferior. However, many acknowledged that although this might be a perception, when they explored this thinking they could rationalise that just because a product is cheaper it does not equate to inferiority.

*“A lot of people say if it is cheaper then it is not as good as the most expensive one.” (parent)*

*“Just because it is cheaper doesn't mean that it is inferior.” (parent)*

Two families suggested switching adult patients prior to paediatric patients to help mitigate some of these concerns.

*“If you want to change, change the adults but don't change the children” (parent)*

### Benefits

Almost all respondents identified cost savings as a benefit of switching to a biosimilar (of note, this was mentioned in the pharmacy letter and prompting questions).

*“I think it is actually probably really good because I do not want the NHS to like not work so I think it is really good that we are going to a cheaper version.” (patient)*

Others identified benefits including greater accessibility to the drug. Increased access felt important for some who remember the challenges of getting biological medication for their child and the impact that doing so had on their lives. Some families highlighted that cost savings could mean that resources could be used elsewhere.

*“Now that there is somebody else out there they might not have to fight to get the drug that their kids deserve. It is good.” (parent)*

Many patients were theoretically willing to accept the risk of possible side-effects for the good of other patients. Patients felt that research data would be another by-product of the switch and were generally positive about contributing towards this.

*“The competition, you have got more opportunities for people to … explore and push the boundaries a bit more in terms of research.” (parent)*

### Equivalence

Although there were anxieties about a biosimilar medication being inferior, a significant majority of respondents expressed that they thought the biosimilar medication would probably be similarly safe and effective.

*“For me, it is the same drug but by a different supplier so it doesn't really make any difference so I do not mind at all.” (parent)*

Even respondents who had significant anxieties about inferiority also simultaneously acknowledged there may be no difference in efficacy. This uncertainty was a relatively universal finding.

*“ … as long as it works as well and I guess until we have tried it, we won't feel reassured about that” (parent)*

### Trust in clinical staff

For many respondents, their anxieties and concerns were mitigated by their faith in their medical team.

*“They wouldn't let you have something that's not going to work.” (parent)*

Even families who were very unhappy about the switch did not blame the medical team and felt that the decision was out of the hands of clinical staff. Frustration regarding the forced switch was an uncommon finding and was generally directed towards the pharmacy, the hospital trust administrators and the NHS.

Most patients had informally heard about the upcoming switch through their treating team, disease support groups or social media. Feedback on the hospital trust pharmacy letter was generally very positive although several patients suggested that a phone call or face to face notification by a familiar member of the treating team may be more effective in the first instance.

## Discussion

To our knowledge this is the first study exploring perceptions on switching to biosimilar medications among paediatric patients and their parents. Achieving data saturation was not a prospective target although clear thematic patterns emerged. While there were minor differences in the specific coding nomenclature and category structure, the two separate analyses identified very similar themes despite separate areas of expertise among the analysts.

The researchers anticipated that the major concerns would be regarding the safety and efficacy of these new mediations. While these issues arose, the overall sentiment was that things would probably be fine and most families were understanding. Patients’ uncertainties appeared to be placated by their trust in the treating team. Altruism was a prominent finding with most families happy to accept the uncertainties of switching for the greater good of the healthcare system.

The much more prevalent concerns had little to do with the actual medication. Patients in particular were most worried about the type of medication administration device, the presence or absence of a stinging preservative and the colour of the medication and packaging. Views were strongly shaped by previous experiences; this was particularly apparent with concerns about the colour of the new product. By the nature of these patients being on adalimumab they will all have used methotrexate (a yellow liquid often with prominent yellow branding and administration devices) in the past or as part of their current treatment regimen. Many families who raised this concern explicitly referred to their previous experience with methotrexate, its associated side-effects and the psychological sequelae from this.

‘Failed switches’ are not uncommon among adults undergoing non-medical monoclonal antibody biosimilar switching with the nocebo factor playing a significant role [20]. Previous experience and expectation significantly affect noxious symptoms attributable to the nocebo affect [25]. Effective communication strategies have been identified as vital in minimizing this phenomenon among adult patients [26, 27]. The themes identified in our study may assist with proactively addressing patient expectations during medication counselling and patient education programs in order to minimise the incidence of failed switching and contribute to an improved patient experience.

It’s known that clinicians often disagree with non-medical switching and feel that patients should be given a choice [28]. Nonetheless, it is important to portray a positive message despite any clinician opinions and biases. We have outlined recommendations on the basis of existing adult literature and the observations from this study (Table 2).

**Table 2:** Recommendations for non-medical paediatric biosimilar switching

Recommendations for non-medical paediatric biosimilar switching	
- Adequate biosimilar teaching for all staff - A coherent message from all members of the team (medical, nursing, pharmacy, others) - Written and verbal patient education prior to switching - Disease and medication specific information to be provided to families - Honest and balanced yet positive framing of information - Detailed information on practical aspects of the biosimilar including device types, additives (including preservatives) and delivery logistics - Information to be written and endorsed by familiar treating team members rather than anonymous administrators/pharmacy	

This study is not without limitations. Participants’ responses would be influenced by multiple local factors, through both informal (discussions with the treating team, local media, social media, support groups) and formal (pharmacy letter) means. Participant bias may have contributed to the positive outlook. Purposive selection is subject to selection bias although this was partially offset by the use of supplementary random sampling for the majority of patients. The data is also limited to a specific medication used among a single disease at one centre. Despite these factors we believe that the findings are generalisable to other healthcare settings and other chronic medical conditions treated with biologics and therapeutic proteins.

## Conclusions

With significant financial strain across many healthcare settings, non-medical biosimilar switching is likely to be an increasingly frequent occurrence. We hope that the findings and recommendations from this study may help clinicians assist their patients through this process.

## Additional files

Additional file 1: Prompting questions. (DOCX 23 kb)

Additional file 2: Pharmacy letter. (DOCX 23 kb)

## Data Availability

The datasets generated and/or analysed during the current study are not publicly available to protect individual privacy but are available from the corresponding author on reasonable request.

## Abbreviations

COREQ: Consolidated Criteria for Reporting Qualitative Research
JIA: Juvenile Idiopathic Arthritis
NHS: National Health Service
SUN: Standardization of Uveitis Nomenclature
