# Supplementary material for: Same but different? A thematic analysis on adalimumab biosimilar switching among patients with juvenile idiopathic arthritis
Source: Pediatr Rheumatol Online J. 2019 Oct 4;17:67. doi: 10.1186/s12969-019-0366-x (PMC6778384; doi:10.1186/s12969-019-0366-x)
Supplement: Supplementary file 1 — Prompting questions. (DOCX 23 kb) [file 12969_2019_366_MOESM1_ESM.docx]

Question one: How old are you?

Question two: Why do you come to the children’s rheumatology department and what is your (child’s) diagnosis?

Question three: How has adalimumab affected the management of your (child’s) condition?

Question four: The initial version of adalimumab was subject to patent; however this recently expired. The NHS is in the process of transitioning patients across to generic, equivalent versions of this medication also known as biosimilars. This should lead to significant savings across the NHS was having the same effect on managing illnesses. How do you feel about this decision?

Question five: How do you personally feel about switching to a biosimilar?

Question six: Do you have any concerns about switching?

Question seven: Can you see any potential benefits of switching?

Question eight: How did you first hear that the hospital was switching adalimumab to a biosimilar?

Question nine: How did you feel about the way you informed about the change to a biosimilar?

Question ten: Can you think any ways that would make the transition to biosimilars easier for patients and families?
