# Supplementary material for: Same but different? A thematic analysis on adalimumab biosimilar switching among patients with juvenile idiopathic arthritis
Source: Pediatr Rheumatol Online J. 2019 Oct 4;17:67. doi: 10.1186/s12969-019-0366-x (PMC6778384; doi:10.1186/s12969-019-0366-x)
Supplement: Supplementary file 2 — Pharmacy letter. (DOCX 23 kb) [file 12969_2019_366_MOESM2_ESM.docx]

Dear [patient name],

I am writing to you on behalf of the Paediatric Rheumatology team as you are currently receiving a biological medication called adalimumab (also known by its brand name, [bio-originator]).

**Biosimilar available**

Until recently, there has only been one supplier of adalimumab in Europe but, following several years of development, a number of other companies have now been licensed to produce the drug (called adalimumab biosimilars). These have been fully evaluated in clinical studies involving many hundreds of adult patients and been shown to have the same effectiveness and side effect profile as the original.

Following guidance from NHS England, we are switching all paediatric patients receiving adalimumab ([bio-originator]) to the [biosimilar] brand of adalimumab over the next few months.

**New Device**

[biosimilar] is available as a prefilled pen and a prefilled syringe if you are receiving a dose of 40mg and as a prefilled syringe if you are receiving a dose of 20mg. Training on using the pre-filled pen is available at this link [product website], and there will also be information about how to use each device in the product packaging. Advice on any aspect of the product is available from Pharmacy on [telephone number].

We are organising a home nurse visit for all paediatric patients with their first dose of [biosimilar]. If this is something you do not feel you will need, please contact [delivery service provider]. If you would like training in using the device in advance of this nursing visit we will be providing an optional training session that you are welcome to attend:

**When will the change happen?**

We will deliver the new product [biosimilar], for your next delivery due after the 2^nd^ January 2019. If you have any [bio-originator] brand adalimumab left, this should be used before changing to use [biosimilar]. If you have any queries relating to the delivery of your medicine please contact [delivery service provider].

If you have any concerns or queries regarding, please feel free to discuss with the pharmacy team on [telephone number].

With kind regards,

[Signed by pharmacy director and paediatric rheumatologists]
